# Enhanced Bruton’s tyrosine kinase in B-cells and autoreactive IgA in patients with idiopathic pulmonary fibrosis

**DOI:** 10.1186/s12931-019-1195-7

**Published:** 2019-10-24

**Authors:** Peter Heukels, Jennifer A. C. van Hulst, Menno van Nimwegen, Carian E. Boorsma, Barbro N. Melgert, Jan H. von der Thusen, Bernt van den Blink, Rogier A. S. Hoek, Jelle R. Miedema, Stefan F. H. Neys, Odilia B. J. Corneth, Rudi W. Hendriks, Marlies S. Wijsenbeek, Mirjam Kool

**Affiliations:** 1 Department of Pulmonary Medicine, Erasmus Medical Center, ‘s-Gravendijkwal 230, 3015 CE Rotterdam, The Netherlands; 2 Department of Pharmacokinetics, Toxicology and Targeting, University of Groningen, Groningen, The Netherlands; 3 Department of Pathology, Erasmus Medical Center, Rotterdam, The Netherlands; 4 Promedior Inc, Lexington, MA USA; 5 Department of Pulmonary Medicine, Amphia hospital Breda, Breda, The Netherlands; 6 GRIAC research Institute, University of Groningen, University Medical Center Groningen, Groningen, The Netherlands

**Keywords:** Idiopathic pulmonary fibrosis, B-cells, Auto-reactive IgA, Bruton’s tyrosine kinase, Bleomycin

## Abstract

**Rationale:**

Idiopathic Pulmonary Fibrosis (IPF) is thought to be triggered by repeated alveolar epithelial cell injury. Current evidence suggests that aberrant immune activation may contribute. However, the role of B-cell activation remains unclear. We determined the phenotype and activation status of B-cell subsets and evaluated the contribution of activated B-cells to the development of lung fibrosis both in humans and in mice.

**Methods:**

B-cells in blood, mediastinal lymph node, and lung single-cell suspensions of IPF patients and healthy controls (HC) were characterized using 14-color flow cytometry. Mice were exposed to bleomycin to provoke pulmonary fibrosis.

**Results:**

More IgA^+^ memory B-cells and plasmablasts were found in blood (*n* = 27) and lungs (*n* = 11) of IPF patients compared to HC (*n* = 21) and control lungs (*n* = 9). IPF patients had higher levels of autoreactive IgA in plasma, which correlated with an enhanced decline of forced vital capacity (*p* = 0.002, r = − 0.50). Bruton’s tyrosine kinase expression was higher in circulating IPF B-cells compared to HC, indicating enhanced B-cell activation. Bleomycin-exposed mice had increased pulmonary IgA^+^ germinal center and plasma cell proportions compared to control mice. The degree of lung fibrosis correlated with pulmonary germinal center B-cell proportions (*p* = 0.010, r = 0.88).

**Conclusion:**

Our study demonstrates that IPF patients have more circulating activated B-cells and autoreactive IgA, which correlate with disease progression. These B-cell alterations were also observed in the widely used mouse model of experimental pulmonary fibrosis. Autoreactive IgA could be useful as a biomarker for disease progression in IPF.

## Background

Idiopathic Pulmonary Fibrosis (IPF) is a progressive and ultimately fatal disorder [1, 2]. Although the natural history of patients with IPF varies from rapid progressive to episodes of relative stability for years, the median survival is 3–5 years following diagnosis [3]. Fibrosis in IPF patients is considered the end-result of exaggerated wound repair due to repetitive subclinical epithelial injury, leading to (myo-)fibroblast activation and uncontrolled matrix deposition [4]. Current disease modifying therapy consists of two drugs that slow down progression of fibrosis [5, 6]. Despite all evidence that immunity is also involved in IPF pathogenesis, treatments that modulate inflammation have failed or even had deleterious effects on primary end-points in clinical IPF trials [7]. It is a topic of debate to what extent immunity is a (co-)driver of disease or whether it is merely an epiphenomenon which can correlate with disease severity.

Recently, several lines of evidence suggest a role for B-cell immunity [8, 9]. Firstly, tertiary lymphoid organs (TLOs) have been found in lungs of IPF patients, which persist and accumulate during disease progression [10, 11]. TLOs harbor a distinct T-cell and B-cell zone, and attraction of B-cells into TLOs depends on CXCL13 [12]. CXCL13 was reported to be elevated in lungs and serum of patients with IPF [12, 13]. Secondly, over the past two decades several specific auto-antibodies have been identified in IPF^8^, likely involving local production of (auto)-antibodies in lung TLOs. Autoreactive antibodies, recognizing pulmonary proteins, may also contribute to repeated lung injury [8]. Thirdly, elevated serum B-cell activating factor (BAFF) was indicative for worse disease outcome in IPF patients [14].

A role for B-cell immunity in IPF may be especially relevant because new therapies that (only) target B-cells or decrease antibody production are effective in other B-cell-mediated interstitial lung diseases and possibly in acute exacerbations of IPF [15–17]. Whether dual treatment of available anti-fibrotic drugs with anti-inflammatory therapies targeting B-cell activation is beneficial in IPF is currently unknown. Hence, we need a better understanding of B-cell profiles in blood and especially in lungs of IPF patients.

Therefore, we performed an in-depth analysis of B-cell phenotype and activation status in blood, mediastinal lymph nodes (LN), and lungs of IPF patients and controls. We analyzed total and/or autoreactive antibodies in plasma and lungs of IPF patients and identified immunological biomarkers for disease progression. Finally, we investigated B-cell subsets in a bleomycin-induced pulmonary fibrosis mouse model.

## Methods

### Study design and subjects

Human lung and Lymph Node (LN) tissue were collected from patients with IPF undergoing lung transplantation. IPF was diagnosed according to current guidelines of the ATS/ERS/JRS/ALAT [1, 18]. All explanted lungs fulfilled pathological criteria for an usual interstitial pneumonia (UIP). As a control, lung tissue was obtained from volume reduction procedures during transplantation upon size mismatch of oversized donor lungs or residual material obtained during surgery for pulmonary tumors. Control residual lung tissue was taken at least > 3 cm from the tumor. For this, only patients with a normal pulmonary function test or mild airflow obstruction (GOLD 1) without emphysema were selected. Control LN were collected from lung transplantation donors. Blood samples were obtained after patient’s informed consent. All patient and healthy subject characteristics are shown in Table 1. The Medical Ethical Committee of the Erasmus MC Rotterdam approved this study (METC 2012–512) and gave consent for collection of residual material of explanted lungs and LN.

**Table 1:** Patient and healthy subject characteristics

	Blood		Lung		MLN	
	HC (*n* = 21)	IPF (*n* = 27)	Control (*n* = 9)	IPF (*n* = 11)	Control (*n* = 13)	IPF (*n* = 10)
Age, years (95% CI)	56 (52–61)	70 (67–73)	50 (56–66)	59 (55–63)	59 (56–61)	58 (53–61)
Gender: M/F (n, (%))	12 (57%) /9	19 (70%)/8	5 (55%)/4	9 (81%)/2	8 (62%)/5	9 (90%)/1
*Diagnosis*						
-MDD: IPF/Prob IPF/Pos IPF(n, (%))		22 (81%)/5/0		11 (100%)/0/0		10 (100%)/0/0
PA obtained (%)		22		100		100
*History*						
-Cardiovascular disease (n, (%))*	0	14 (51%)	3 (33%)	2 (18%)		2 (20%)
-Auto-immune disease (n, (%))^#^	0	1 (3%)	1 (11%)	0 (0%)		0 (0%)
*PFT*, *percentage predicted* (95% CI)						
-TLCO		47 (42–53)	86 (76–95)	33 (27–39)		34 (27–40)
-FVC		77 (72–83)	108 (95–121)	53 (45–60)		52 (44–60)
-FEV1/FVC			69 (63–73)			
*COPD* (n) (stage)		0	3 (all GOLD1)	0		0
*Smoking*, n (%)						
-never		5 (19%)	2 (22%)	2 (18%)		2 (20%)
-former		22 (81%)	7 (78%)	9 (82%)		8 (80%)
-current		0 (0%)	0 (0%)	0 (0%)		0 (0%)
*Medication*						
-prednisone use: (n, (%))		8 (30%)	0 (0%)	4 (36%)		3 (30%)
*dose (mg/day)*						
> 10 mg/d		0 (0%)		1 (9%)		0 (0%)
</= 10 mg/d		8 (30%)		3 (27%)		3 (30%)
pa Other immunosuppressive (n, (%))		0 (0%)	0 (0%)	0 (0%)		0 (0%)
PO Please Pirfenidone (n, (%))		0 (0%)		2 (18%)		2 (20%)
Nintedanib (n, (%))		1 (3%)		3 (27%)		3 (30%)

* *IPF patients (blood)*: Hypertension (n = 9), ischemic cardiac disease (*n* = 5), or transient ischemic attack (n = 2), *Control lung*: Hypertension (*n* = 3), Cerebro Vasculair Accident (n = 2)

*IPF lung and LN*: Hypertension and transient ischemic attack (n = 1), Hypertension (n = 1)

# *IPF patients (blood)*: colitis ulcerosa (not active and no immunomodulating medication) (n = 1)

*Control lung*: Rheumatoid arthritis (not active and no immunomodulating medication) without lung involvement or anti-CCP antibodies (*n* = 1)

Abbreviations: MLN = mediastinal lymph node, MDD = multidisciplinary diagnosis, PFT = pulmonary function test, TLCO = carbon monoxide transfer factor, FVC = forced vital capacity, FEV1 = forced expiratory volume in one 1 s

### Human tissue and blood processing

Fresh lung and LN tissues were stored in cold phosphate buffered saline (PBS) and processed within 24 h following transplantation or resection.

Lung resection specimens were rinsed with PBS to remove residual blood. After mincing the lung, specimens were enzymatically digested in digestion medium (Life Technologies) with 10μg Liberase (Roche, LiberaseTM) and 40 Units of DNA-se (Roche, DNase I recombinant, RNase-free) for 30 min in a humidified incubator at 37 °C while gently shaking the samples. The remaining cell debris was removed by passing the cells through a 100 μm-diameter disposable cell mesh filter. Fresh LN were separately past through a 100 μm-diameter disposable cell mesh filter. Single cell suspensions specimens were washed in Roswell Park Memorial Institute medium (RPMI) with 5% fetal calf serum and centrifuged for 10 min at a speed of 400×g. Lung single cell suspensions were also subjected to red blood cell lysis, washed and counted. Finally, all samples were aliquoted and cryopreserved .

Blood samples were collected in ethylenediaminetetraacetic acid (EDTA) tubes (BD Vacutainer K2E). Peripheral blood mononuclear cells (PBMCs) and plasma were obtained, processed and stored according to standard protocols [19].

PBMCs and single-cell suspensions were stained for extra- and intracellular markers described in Additional file 10: Table S1**.** PBMCs and single cell suspensions were stained with antibodies for extra- and intracellular markers described in Additional file 10: Table S1. To control for non-specific labeling Fc-block (Anti-Mouse CD16/CD32 Fc Block) was used. Fixable Viability Dye eFluor 506 (eBiosciences) was applied as a live-dead marker. In short, cells for the B-cell staining were incubated in FACS buffer (PBS, 0.25% BSA, 0.5 mM EDTA, 0.05% NaN3 sodium azide) and for the T-cell staining in MACS buffer (0.5% BSA + 2 mM EDTA in PBS) with fluorescent antibodies for 60 min at 4 °C using methods recommended by the manufacturers. Cells for the T-cell staining underwent a second extracellular incubation step for antibodies with Brillaint Viotet (BV) conjungates in BV-bufferin Brilliant Stain-buffer (BD Biosciences, cat#563794). After fixation and permeabilization (2% paraformaldehyde solution and 0,5% saponin (Quillaja Bark, Sigma cat#S7900) cells were incubated with the Bruton’s tyrosine kinase (BTK)-antibody or Isotype control in Saponine buffer for 60 min at 4 °C (only B-cell staining). Biotinylated antibodies were visualized with streptavidin-BV650. Cells were measured on a LSRII Flow cytometer (BD Biosciences). We analyzed a minimum of 200,000 alive cells for blood samples and 100,000 alive cells for the lung tissue samples for cytometric analysis. Data was analyzed by FACS Flow-Jo software. To compare mean fluorescence intensity (MFI) values between experiments, identical control samples (*n* = 3) were measured in each (subsequent) experiment for standardization.

### Immunohistochemistry

Immunohistochemical analyses were performed according to standard procedures [19]. Antibodies used are listed in Additional file 10: Table S1.

### Self-reactive and total immunoglobulin (Ig) G, IgM and IgA

HEp-2 analyses and enzyme-linked immunosorbent assay (ELISA) on plasma samples were performed according to standard procedures. Serum samples (1/50 diluted) of IPF patients and healthy controls (HC) were incubated for 1 h on Kallestad human epithelial cell (HEp-2) slides (Bio-Rad Laboratories). As detection antibodies Ig F (ab’)2 fragments were applied to the HEp2 slides (Additional file 10: Table S1). The fluorescence intensity of HEp2 slides was evaluated using a LSM 311 META confocal fluorescence microscope (Zeiss) and LSM Image Browser Version 4.2.0.12 software (Zeiss) in an automated and thus independent manner. The fluorescence intensity was corrected for number of cells per slide. A positive HEp-2 result was set on 2SD above the mean of the HC for each immunoglobulin subtype.

### Mice

Mice were bred and kept under specified pathogen-free conditions in the Erasmus MC experimental animal facility. All experimental protocols have been reviewed and approved by the Erasmus Medical Center Committee of animal experiments. Procedures of mouse experiments are described in Additional methods 1.

### Statistics

Statistical analysis was performed using IBM SPSS Statistics 21 and GraphPad Prism 6 software. For calculating the level of significance of differences between groups we used the Mann-Whitney U test. Correlation coefficients were calculated using Spearman’s rank method. *P* values < 0.05 were considered significant. Flow cytometry data is either represented as percentage population or as MFI.

## Results

### Alterations in B-cell subsets in blood, LN and lungs of IPF patients

**Fig. 1:**
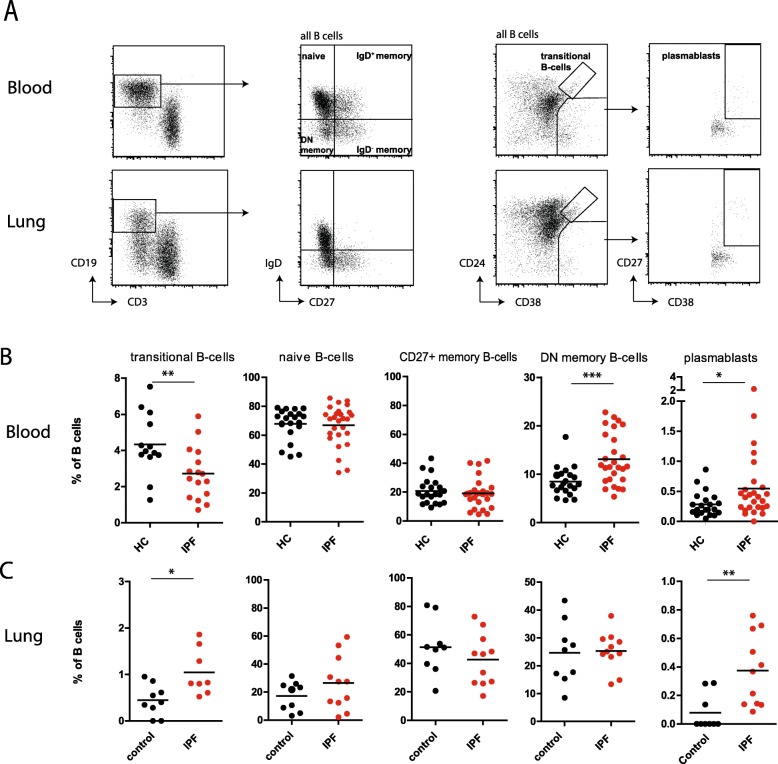
Alterations in B-cell subsets in blood, LN and lungs of IPF patients. ((**a**) Representative gating strategy for identification of B-cell subsets in blood and single cell suspensions of lungs. Naïve B-cells (CD19^+^IgD^+^CD27-) IgD^+^ and IgD^−^ memory CD27^+^ B-cells, double negative (DN) B-cells (CD19^+^IgD^−^CD27^−^), transitional B-cells (CD19^+^CD24^+^CD38^+^), and plasmablast (CD19^+^CD38^+^CD27^+^) were identified. (**b**) c Percentage of circulating B-cell subsets of total B cells in HC (*n* = 21) and IPF patients (n = 27). (**c**) Proportion of B-cell subsets of total B-cells in single cell suspensions of control lungs (*n* = 9) and explanted IPF lungs (*n* = 11). Non-parametric two-tailed Mann-Whitney test was used. Data are expressed as mean and dots represent individual patient values. * P < 0.05 ** *P* < 0.01 *** P < 0.001

We first evaluated different B-cell subsets in blood, explanted lungs, and LN of IPF patients. We quantified naïve B-cells (IgD^+^ CD27^−^), IgD^+^ and IgD^−^ memory CD27^+^ B-cells, double negative (DN) B-cells (IgD^−^CD27^−^), transitional B-cells (CD19^+^CD24^+^CD38^+^), and plasmablasts (CD19^+^CD38^+^CD27^+^), following the gating strategy in Fig. 1 A.

The numbers and percentages of total B-cells in blood, LN, and lung were similar between IPF patients and controls (Additional file 1: Figure S1). The proportions of naïve B-cells were higher in blood than in lung, but did not differ between control and IPF (Fig. 1 B /C). In the circulation, IPF patients had a lower frequency of transitional B-cells, but significantly higher proportions of DN memory B-cells and plasmablasts compared with HC (Fig. 1 B). IPF lungs harbored higher proportions of transitional B-cells and plasmablasts compared to control lungs (Fig. 1 C). No alterations in B-cell subsets were observed between IPF LN and control LN (Additional file 2: Figure S2).

### Augmented proportions of IgA-expressing memory B-cell subsets

**Fig. 2:**
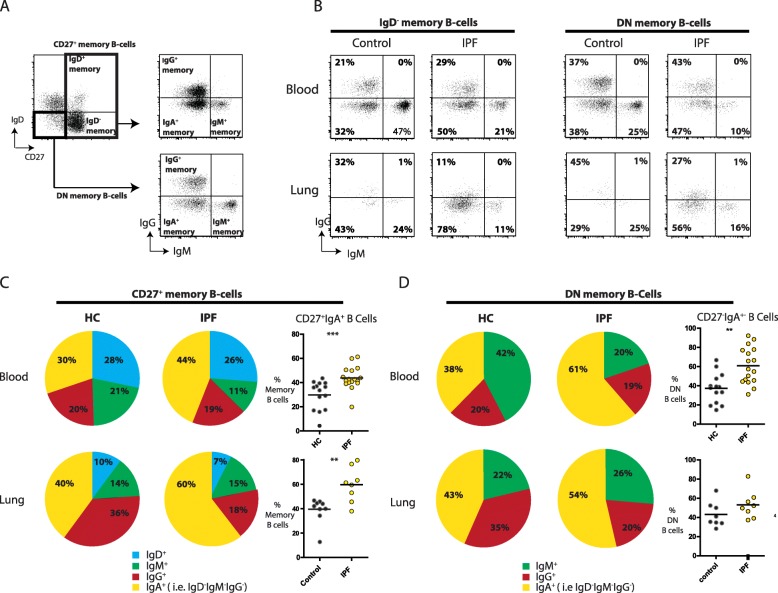
Augmented proportions of IgA-expressing memory B-cell subsets. (**a**) Representative gating strategy for the identification of immunoglobulin surface expression (IgM or IgG) on IgD^−^ memory B-cells CD19^+^CD27^+^IgD^−^) or double negative (DN) B-cells (CD19^+^CD27^−^IgD^−^). (**b**) Gating strategy as described in panel A for a representative blood and lung sample for a control and IPF patient. (**c**; left) Pie chart showing the distribution of surface expression of IgD, IgM, IgG, and IgA on CD27^+^ memory B-cells depicted with mean percentage; (**c**: right) Proportions of IgA^+^ memory B-cells of total CD27^+^ memory B-cells in blood and lung samples. (D; left) Distribution of immunoglobulin expression of IgM, IgG and IgA on DN memory B-cells depicted in a pie-chart with mean percentage. (**d**; right) Proportions of IgA^+^ DN memory B cells of total DN B-cells in blood and lung samples Non-parametric two-tailed Mann-Whitney test was used. Data are expressed as mean and dots represent individual patient values. ** *P* < 0.01 *** *P* < 0.001.

We next evaluated surface immunoglobulin (Ig) expression on memory B-cell subsets in blood and single-cell suspensions of explanted IPF lungs. Surface IgM and IgG expression was evaluated on IgD^−^ memory B-cells and DN-memory B-cells (Fig. 2 A) as depicted in a representative blood and lung sample from an IPF patient and a healthy control in Fig. 2 B. The IgD^−^IgM^−^IgG^−^ memory and IgM^−^IgG^−^ DN-memory B-cells were enriched for IgA surface expression (Additional file 3: Figure S3 and Additional file 4: Figure S4). These populations are hereafter called IgA^+^ memory B-cells.

The proportion of IgA^+^ cells within the CD27^+^ memory B-cell fraction was significantly higher in IPF patients in blood (~ 44%, compared to ~ 30% in controls) and in the lung (~ 60% versus ~ 40%, respectively) (Fig. 2 C). Likewise, within in the DN-memory B-cells, the IgA^+^ population was significantly higher in blood of IPF patients compared to controls (Fig. 2 D). Within both memory B-cell populations, this increase of IgA^+^ B-cells was at the expense of IgM^+^ B-cells in blood and at the expense of IgG^+^ B-cells in lungs (both significant) (Fig. 2C, D**;** Additional file 3: Figure S3 and Additional file 4: Figure S4). No differences in surface Ig expression on memory B-cell subsets were observed between explanted IPF and control LN (Additional file 3: Figure S3 and Additional file 4: Figure S4)**.**

Summarizing, within the two memory populations in blood and lung, IgA-expressing B-cells were increased in IPF patients and dominant over B-cells expressing other Ig subclasses.

### IgA^+^ B cells are present within TLO structures of IPF lungs

**Fig. 3:**
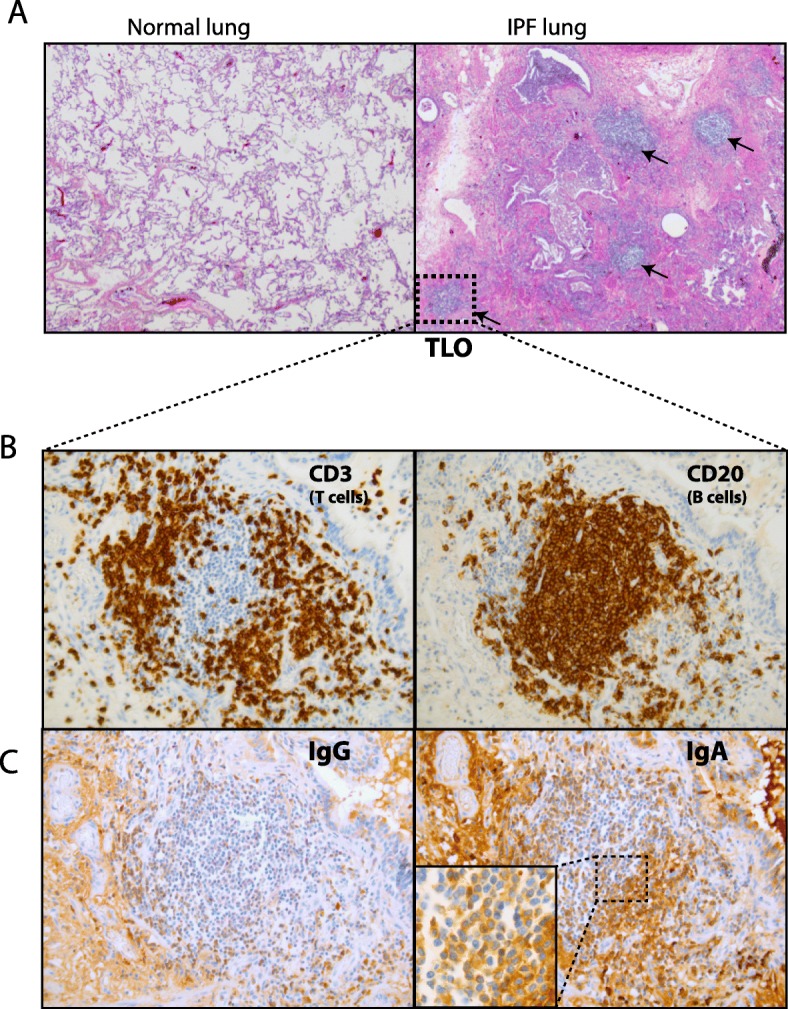
IgA^+^ B cells are present within TLO structures of IPF lungs. (**a**) Hematoxylin and eosin (**h**&**e**) staining of a control lung and IPF lung showing numerous TLOs (black arrows). (**b**) Pulmonary TLOs of IPF lung stained with anti-CD3 (T cells) and anti-CD20 (B-cells). (**c**) Representative images of staining with anti-IgG and anti-IgA. Magnification: 10x (**a**) and 40x (**b** and **c**)

We next examined whether IgA expressing B-cells are present within TLOs found in IPF lungs [11]. In the lungs of our IPF patient cohort numerous TLOs could be detected (Fig. 3 A). We next confirmed that IPF TLOs had a specific organization with segregated T and B-cell zones (Fig. 3 B). Within the B-cell area, IgG staining was weak, however IgA staining was strong (Fig. 3 C). IgA could also be detected outside TLOs, likely reflecting secreted IgA.

Thus, IgA^+^ B-cells were present in TLO in IPF lungs and probably contribute to the local IgA production.

### More activated follicular helper T-cells (Tfh) in IPF lungs

**Fig. 4:**
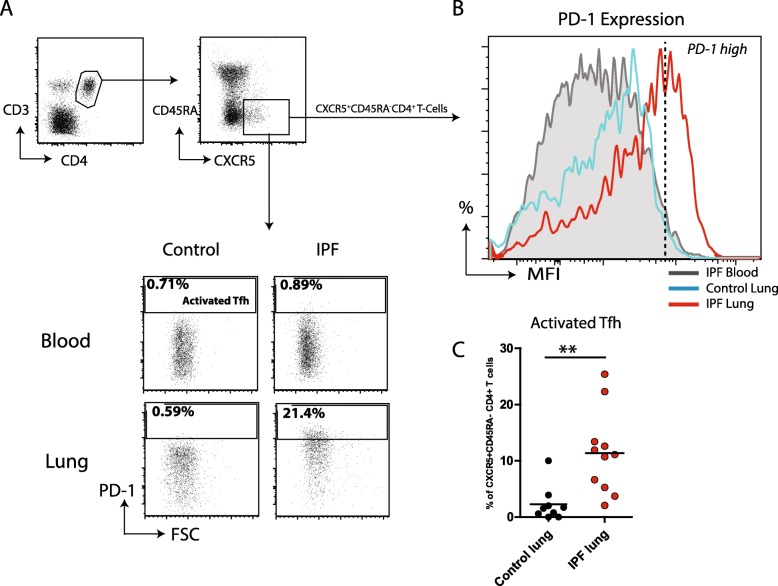
More activated follicular helper T-cells (Tfh) in IPF lungs. (**a**) Representative gating strategy for identification of Tfh (CXCR5^+^CD45RA^−^CD4^+^) and activated Tfh (CXCR5^+^CD45RA^−^CD4^+^PD-1^HI^) as depicted for a representative blood and lung single cell suspension for a control and IPF patient. (**b**) Representative histogram overlay showing PD-1 expression levels of Tfh-cells depicted as MFI for a blood sample of an IPF patient (gray), control lung (blue) and IPF lung (red). (**c**) Activated Tfh-cells as percentage of total Tfh-cells in single cell suspensions of control lungs (*n* = 8) and IPF lungs (*n* = 11). Non-parametric two-tailed Mann-Whitney test was used. Data are expressed as mean and dots represent individual patient values. ** *P* < 0.01

Active TLOs contain germinal centers (GCs) in which follicular T helper (Tfh) cells provide essential help to B-cells to promote their activation. Tfh-cells, identified as CD3^+^CD4^+^CXCR5^+^CD45RA^−^ cells, were evaluated for the expression of PD-1, which is a reliable marker for activated Tfh-cells (Fig. 4 A) [20]. The proportions of Tfh-cells as percentage of CD4^+^ T-cells did not differ between HC and IPF patients (blood and lung, data not shown). PD-1 expression was low in IPF and control blood, but was higher in IPF lung tissue (Fig. 4 A, B). Hereby the proportions of activated PD-1^high^ Tfh-cells in IPF lungs was significantly higher compared to control lungs (Fig. 4 C), consistent with the presence of active GCs in pulmonary TLOs.

### Autoreactive IgA and IgG levels are higher in IPF and autoreactive IgA correlates with disease progression

**Fig. 5:**
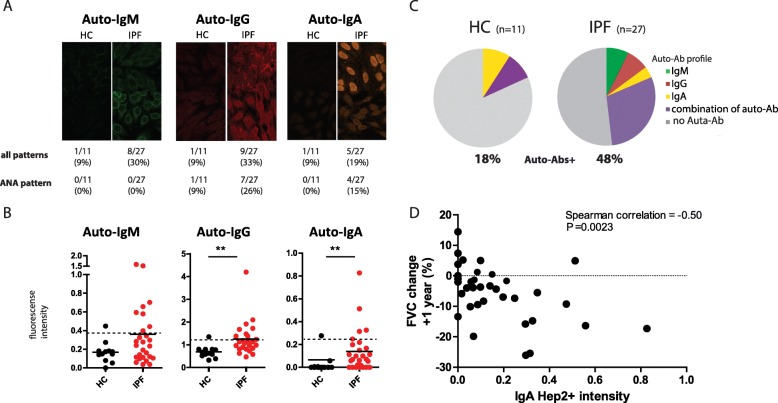
Autoreactive IgA and IgG levels are higher in IPF and autoreactive IgA correlates with disease progression. (**a**) Total IgM, IgG, and IgA in plasma of IPF patients and HCs. (**b**) Representative staining pattern of human epithelial cells (HEp)-2 slides with plasma of HC or IPF patients. First row describes the number and percentage of patients with a positive staining and second row number and percentage of patients with a specific antinuclear antibody (ANA) staining pattern. (**c**) Indirect quantification of auto-reactive immunoglobulins levels depicted as fluorescence intensity for autoreactove-IgM, IgG and IgA assessed with HEp-2 staining. The fluorescence intensity of HEp2 slides was evaluated in an automated and thus independent manner. The fluorescence intensity was corrected for number of HEp-2 cells per slide. A positive HEp-2 result was set on 2x standard deviation above the mean of the HC for each immunoglobulin subtype (see also additional methods 1) (**d**) Pie-chart of percentage of HC or IPF patients with detectable autoreactive antibodies and subclass (IgM, IgG and IgA (or combination)). (**e**) Increased fluorescence intensity for plasma autoreactive IgA correlate with decline in forced vital capacity (FVC) over 1-year period. Data of 12 IPF patients (with multidisciplinary diagnosis (MDD) of definitive or probable IPF) were used from our original cohort of 27 patients. 3 patients in the original cohort died within one year and could not be used for this analysis. Correlation coefficients were calculated using Spearman’s rank method. Non-parametric two-tailed Mann-Whitney test was used. Data are expressed as mean and dots represent individual patient values. * *P* < 0.05 ** *P* < 0.01

Total IgG and IgA – but not IgM – was significantly higher in plasma of patients with IPF compared with HC (Additional file 5: Figure S5). To explore the presence of auto-antibodies in plasma from IPF patients, we evaluated HEp-2 staining patterns and their fluorescence intensity for IgM, IgG and IgA. The staining patterns revealed the presence of autoreactive IgG and IgA, mainly recognizing nuclear antigens and showing a homogeneous staining across the nucleoplasm. Quantification of the autoreactive IgG and IgA fluorescence intensities, reflecting their plasma concentrations, revealed a significant increase in plasma of IPF patients compared to HC (Fig. 5 B). Also, the proportion of IPF patients harboring autoreactive IgM, IgG, or IgA was increased compared to HC (Fig. 5 A, B). There was no correlation between total and autoreactive IgG/IgA levels (Additional file 5: Figure S5). In ~ 48% of the IPF patients detectable autoantibodies were present versus in 18% of the HC, being IgM, IgG, or IgA **(**Fig. 5 C). Interestingly, within the IPF patients that had autoreactive antibodies, a majority (~ 62%) had autoantibodies of multiple isotypes.

We next evaluated whether levels of circulating auto-antibodies correlated with measures of disease progression. No correlation was observed between auto-reactive IgG or IgM with 1-year forced vital capacity (FVC) change (Additional file 6: Figure S6). However, higher amounts of autoreactive IgA correlated with an increased decline of FVC (Fig. 5D).

In summary, about half of the IPF patients analyzed had circulating IgM, IgG or IgA anti-nuclear auto-antibodies (ANAs), whereby autoreactive IgA correlated with disease progression.

### Increased Bruton’s tyrosine kinase expression levels in B cells of IPF patients

**Fig. 6:**
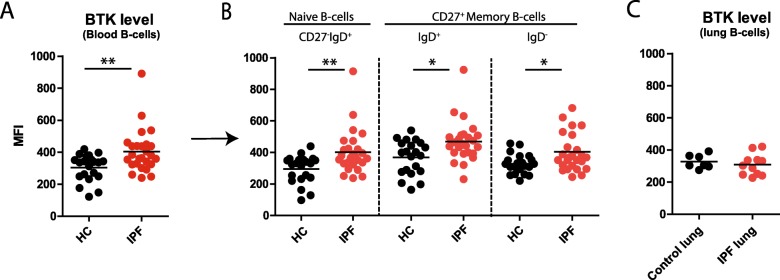
Increased Bruton’s tyrosine kinase (BTK) expression levels in B cells of IPF patients. (**a**) Mean fluorescence intensity (MFI) of BTK in all circulating B-cells (CD19^+^) and (**b**) naïve B-cells (CD27^−^IgD^+^), IgD^+^ memory B-cells (CD27^+^IgD^+^) and IgD^−^ memory B-cells (CD27^+^IgD^−^) of HCs and IPF patients. (**c**) BTK expression (MFI) in pulmonary B-cells of control lungs and IPF lungs. Non-parametric two-tailed Mann-Whitney test was used. Data are expressed as mean and dots represent individual patient values. * P < 0.05 ** P < 0.01

Given the reported increased protein expression of the signaling molecule Bruton’s tyrosine kinase (BTK) in B-cells from patients with various autoimmune disorders, such as rheumatoid arthritis, Sjogren’s Syndrome and vasculitis [21], we next evaluated BTK expression in circulating B-cells by intracellular flow cytometry. We observed higher BTK protein levels in IPF compared to HC (Fig. 6 A). Specifically, naïve B-cells showed the most pronounced enhanced BTK expression in IPF patients (Fig. 6 B). We found no difference in BTK levels between treatment-naïve patients and patients using low-dose prednisone (data not shown). In lungs and LN of IPF patients, BTK expression in B-cell subsets was similar to controls (Fig. 6 C).

Taken together, our data demonstrate that in blood of IPF patients, BTK expression in B-cells was higher than in HC.

### Bleomycin-induced IgA+ induction and GC B-cells correlate with fibrosis scores in a mouse model

**Fig. 7:**
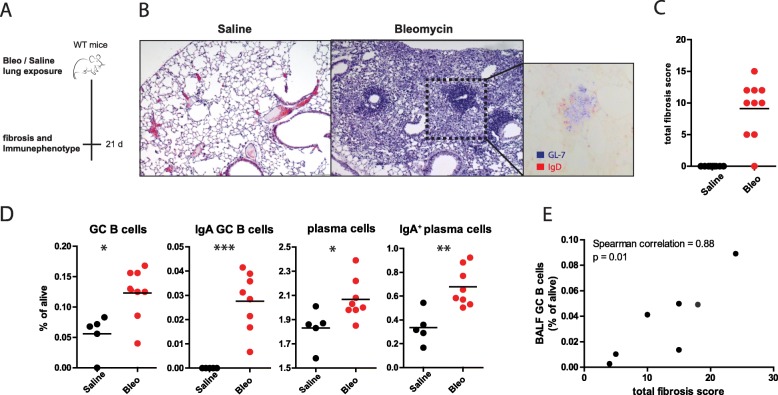
Bleomycin-induced fibrosis promotes germinal center IgA^+^ B-cells and IgA^+^ plasma cells. (**a**) Mice were sacrificed 21 days after saline or bleomycin exposure and analyzed for fibrosis indices and inflammation. (**b**) Representative hematoxylin and eosin (**h**&**e**) staining of cryo-sections of lung tissue after PBS or bleomycin exposure. Histological analysis revealed that pulmonary fibrosis was present in mice 21 days after bleomycin exposure as shown by typical characteristics including thickening of alveolar walls with or without obvious damage and formation of fibrous bands. The dashed square shows a probable TLO structure, which showed a center GL-7-positive cells (present on GC B-cells) surrounded by IgD- positive naïve B-cells . (**c**) Total fibrosis score (TFS). The TFS is the product of the Ashcroft scale and level of lung involvement (see additional methods 1). ((**d**) Proportion of GC B-cells (CD19^+^CD95^+^IgD^low^), IgA GC B-cells (CD19^+^CD95^+^IgD^low^IgA^+^), plasma cells (CD19^low^CD138^+^), and IgA^+^ plasma cells (CD19^low^CD138^+^IgA^+^) of alive cells in lungs. (**e**) Correlation of the proportion of GC B cells of alive cells in bronchoalveolar lavage fluid (BALF) and TFS. Non-parametric two-tailed Mann-Whitney test was used. Correlation coefficients were calculated using Spearman’s rank method. Data are expressed as mean and dots represent individual values of 4–7 mice per group and representative of 2 or more independent experiments. * P < 0.05 ** P < 0.01 *** *P* < 0.001

To explore if changes seen in human B-cell subsets would also occur in a well-established mouse model for fibrosis, we subjected C57Bl/6 mice to bleomycin or saline (control group). Histological analysis revealed that pulmonary fibrosis was present in mice 21 days after bleomycin exposure (Fig. 7 A). Lungs of bleomycin-exposed mice contained inflammatory aggregates with a core of GL-7+ GC B-cells and a ring of IgD^+^ naïve B cells (Fig. 7 A). Blinded histopathology scoring confirmed that the total fibrosis score (TFS) was elevated in the bleomycin-exposed group (Fig. 7 B). Next, we evaluated B-cell subsets in lungs by flow cytometry (Fig. 7 C and Additional file 7: Figure S7). The frequency of pulmonary GC B-cells and plasma cells of alive cells were significantly higher in mice exposed to bleomycin compared to saline-exposed mice. Within GC B-cells or plasma cells respectively, the proportions of pulmonary IgA^+^ GC B-cells and IgA^+^ plasma cells were increased (Additional file 8: Figure S8). In bleomycin-exposed mice, the TFS and the frequency of bronchoalveolar lavage fluid (BALF) GC B-cells showed a positive correlation (Fig. 7D).

In summary, mice subjected to bleomycin-induced pulmonary fibrosis showed similar pathobiological changes as in human IPF. The extent of fibrosis correlated with the proportion of BALF GC B-cells.

## Discussion

Our study assessed B-cell subsets and their activation status in IPF lungs and LN by 14-color flow cytometry. We observed that IPF patients had increased plasmablasts and IgA^+^ memory B-cells in blood, intense IgA staining in lung TLOs, and more serum IgA antibodies recognizing nuclear self-antigens. Higher autoreactive IgA levels correlated with an increased decline of FVC. Furthermore, we showed that circulating IPF B-cells had elevated BTK expression, which could contribute to a loss of immune tolerance and development of autoimmunity in IPF (Additional file 9, graphic summary). In a bleomycin pulmonary fibrosis mouse model, we observed the induction of IgA^+^ GC B-cells and IgA plasma cells, indicating similarities with human pathobiology. The bleomycin model also showed that proportions of BALF GC B-cells correlated with the extent of fibrosis.

The increased numbers of TLOs in lungs of IPF patients, together with elevated serum CXCL13 and BAFF, indicate involvement of B-cells in IPF [11–14]. The decrease in circulating transitional B-cells, together with an increase of B cells in the lungs of IPF patients, suggests migration from the blood towards the lungs. Transitional B-cells, which are immature B cells that have recently emigrated from the bone marrow, are possibly attracted to pulmonary TLO in IPF patients by CXCL13. We confirmed that plasmablasts are increased in blood [14], and importantly, also in IPF lungs. Furthermore, DN-memory B-cells are increased blood of IPF patients. This is particularly interesting as DN-memory B-cells activate telomerase when stimulated and are thought to be age-related exhausted memory B-cells [22, 23]. As both telomerase dysfunction and immune exhaustion have been implicated in IPF pathogenesis [24–26], this B-cell subset would be an interesting topic for further research.

Repetitive alveolar epithelial injury exposes our immune system to self-antigens, increasing the risk of induction of autoreactive antibodies. Transforming growth factor-β, a key profibrotic cytokine, promotes IgA class-switch recombination of B-cells [27, 28]. Serum IgA concentrations at the time of IPF diagnosis have been shown to be a predictor of survival [29]. In our patient cohort, plasma levels of total IgA and autoreactive IgA (mostly recognizing ANA) were also enhanced and specifically autoreactive IgA correlated with disease progression. Previous studies showed that autoantibodies found in IPF patients are often of the IgA subclass [8, 30]. Although (autoreactive) IgG antibodies are also implicated in IPF pathogenesis, we did not find a correlation with lung function decline [8, 9]. Autoreactive IgA may promote lung fibrosis through fibroblast proliferation and extracellular matrix protein production [31, 32]., This leads to a detrimental feedforward loop as the profibrotic milieu in IPF lungs promotes IgA class switching in B-cells.

BTK is a key molecule in BCR signaling and increased BTK expression in B-cells promotes the development of autoreactive B-cells [21]. Small-molecule BTK inhibitors have been proven effective in the treatment of autoimmune diseases in preclinical studies [21, 33]. In IPF patients, BTK expression was increased in circulating B-cells, especially in naïve B-cells. This might either reflect global B-cell activation due to pro-inflammatory micro-environments or pathogenic B-cell activation that directly contributes to autoimmune pathology. Activated Tfh-cells, which are present in IPF lungs, have the capacity to engage with B-cells in the GCs of pulmonary TLOs and might promote autoreactive B-cell development [34]. Interestingly, mice with B-cell specific overexpression of BTK develop an autoimmune phenotype, together with increased Tfh-cell proportions [35]. These Tfh-cells might be induces by increased CD80 and CD86 expression on B-cells, which provide costimulatory signals for T-cell activation [35]. Consequently, our findings in IPF patients that (i) increased proportions of circulating plasmablasts and (IgA-)memory B-cells with (ii) enhanced BTK expression, (iii) augmented pulmonary Tfh-cell activation, and (iv) enhanced plasma autoreactive IgA are present indicate an important role for B-cells in IPF pathogenesis.

To investigate whether the same B-cell alterations would occur in a well-established model for pulmonary fibrosis, mice were exposed to bleomycin. Indeed, bleomycin exposure increases lung IgA^+^ GC B-cells and IgA^+^ plasma cells. The degree of pulmonary fibrosis correlated with the frequencies of GC B-cells in BAL fluid. This suggests that TLO formation with an active GC and the presence of IgA^+^ GC B-cells reflects the degree of pulmonary fibrosis. The local pro-fibrotic environment together with the pulmonary mucosal environment is most likely responsible for the IgA class-switch recombination.

The bleomycin model deviates from human IPF pathology as fibrosis development is not progressive and it depends on inflammation [36]. Nevertheless, our data show similarities with human B-cell pathobiology. A previous study explored the effects of the irreversible BTK-inhibitor ibrutinib on fibrosis development and unexpectedly exacerbated fibrosis was found [37]. However, the interpretation of this finding is complicated, because ibrutinib is shown to have off-target effects and BTK is also expressed in myeloid lineages [37, 38].

At this moment, it remains unclear if changes in B-cell activation or subsets are the primary culprit for IPF disease onset and/or progression. Nevertheless, changes in B-cell immunology may be a valuable biomarker for disease progression and/or survival, as high BAFF and CXCL13 plasma concentrations, involved in B-cell activation and homing, are predictive for poor survival in IPF patients [13, 14].

Rituximab, an antibody against CD20 which destroys CD20^+^ B cells, is effective in the treatment of several auto-immune diseases [39]. Compared to a historical cohort, an improvement of gas exchange and clinical outcome was observed in IPF patients with an acute exacerbation treated with rituximab, together plasma exchange and intravenous immune globulin [15]. The same experimental therapy is now under investigation in patients with acute exacerbations of IPF in a phase 2 trial (NCT03286556). If rituximab alone could be beneficial in the era of anti-fibrotic drugs is also currently examined and the results of this trial are expected in 2020 (NCT01969409). We believe that rituximab or other B-cell modulating therapies could be of value in the treatment of IPF patients next to ‘anti-fibrotic’ medication. Large landmark trials in the past using broad acting anti-inflammatory molecules in unselected IPF patients have learned that “one size fits all” approach does not always work [7]. Therefore, in our opinion, selection of IPF patients eligible for B-cell modulating therapies should be based on inflammatory biomarkers, preferably those that reflect B-cell (auto-) reactivity or activations status.

Our study has some limitations: First, lungs of diseased patients who underwent surgery, were used as controls. Three of these controls had mild COPD. We cannot rule out that inflammation profiles are affected, as recent literature show an increase of IgA expression and accumulation of B-cells in lung TLO in patients with severe COPD [40, 41]. However, subgroup-analysis showed that the COPD lungs had similar outcomes in B-cell subsets and activation markers compared with other controls (data not shown). Using disease controls could also contribute similar BTK expression in pulmonary B-cells in IPF patient and controls, as the majority of pulmonary B-cells have been activated. Second, no differences in B-cell subsets were observed in LN in our human data. The IPF LN examined in this study came from transplanted IPF patients, indicating end-stage IPF, which could be different in T- and B-cell proportions compared to LNs in early IPF disease. TLOs persist and accumulate during disease progression in IPF lungs, which may suggest that local immune activation becomes more important in end-stage disease [11]. Furthermore, LN enlargement does not necessarily change the proportion of B-cell subsets. Third, the relatively small sample size of human IPF lungs and LN used in our experiments. To the best of our knowledge, our study is the first that assessed B-cell subsets and activation in explanted IPF lungs and LN by detailed flow cytometry profiling and significant differences could be observed. Fourth, potential confounding by subject drop-out in the 1-year FVC change correlation analysis, as 4 patients died within the first year. Finally, two specimens (one IPF blood sample and one control lung) were obtained from subjects with a history of auto-immune disease. These samples were not excluded as (i) both subjects did not use immunomodulating medication, (ii) showed no clinically active disease at the time of sample collection, and (iii) the obtained B-cell data were within the range of the other samples.

## Conclusion

We provide evidence that (autoreactive) B-cells, especially IgA-memory B-cells, are increased in IPF patients, possibly driven by increased intracellular BTK expression. Additionally, autoreactive IgA could be a predictor for FVC decline, however larger validation studies are needed to investigate the potential of autoreactive IgA as a new biomarker in IPF.

There is a need for stratified medicine based on inflammatory biomarkers and profiles to select patients with IPF who may be eligible for co-treatment with anti-inflammatory or immunomodulating therapies next to ‘anti-fibrotic’ medication. Our study provides a rationale for B-cell modulating therapies in selected IPF patients.

## Supplementary information

**Additional file 1: Figure S1.** No changes of proportions of total B-cells between controls and IPF patients. Flow cytometric quantification of total B-cells (CD19^+^) in blood, lungs and lymph nodes (LN) as percentage of alive cells. For blood samples data also depicted as absolute number of B-cells per ml blood. Data are expressed as mean and dots represent individual values

**Additional file 2: Figure S2.** No alterations in B-cell subsets between control lymph nodes (LN) and IPF LN. Flow cytometric quantification of naïve B-cells (CD19^+^IgD^+^CD27-) IgD^+^ and IgD^−^ memory CD27^+^ B-cells, double negative (DN) B-cells (CD19^+^IgD^−^CD27^−^), transitional B-cells (CD19^+^CD24^+^CD38^+^), and plasmablast (CD19^+^CD38^+^CD27^+^) as percentage of alive cells. Data are expressed as mean and dots represent individual values.

**Additional file 3: Figure S3.** Augmented proportions of IgA-expressing CD27^+^ memory B-cell subsets. (A) Representative gating strategy for the identification of surface IgA expression on CD27^+^IgD^−^ memory B-cells. The CD27^+^IgD^−^IgM^−^IgG^−^ memory cells are enriched for IgA^+^ surface expression as depicted for a healthy control (HC) and IPF patient. (B) Flow cytometric analysis of the distribution of surface expression of IgD, IgM, IgG and IgA on CD27^+^ memory B-cells for blood, lungs and lymph nodes for controls and IPF patients. Non-parametric two-tailed Mann-Whitney test was used. ** *P* < 0.01 *** *P* < 0.001

**Additional file 4: Figure S4.** Augmented proportions of IgA-expressing DN memory B-cell subsets. (A) Representative gating strategy for the identification of surface IgA expression on double negative (DN) memory B-cells (CD19^+^CD27^−^IgD^−^). The IgM^−^IgG^−^ DN memory cells are enriched for IgA^+^ surface expression as depicted for a healthy control (HC) and IPF patient. (B) Flow cytometric analysis of the distribution of surface expression of IgM, IgG and IgA on DN memory B-cells for blood, lungs and lymph nodes for controls and IPF patients. Non-parametric two-tailed Mann-Whitney test was used. ** P < 0.01 *** P < 0.001

**Additional file 5: Figure S5.** Increased total IgG and IgA in plasma of IPF patients. (A) total plasma IgM, IgG and IgA levels (μg/ml) for HC and IPF patients. (B) Correlation between total IgG or IgA (μg/ml) and autoreactive IgG or IgA. Indirect quantification of auto-reactive immunoglobulin levels depicted as fluorescence intensity measured on HEp-2 slides. Non-parametric two-tailed Mann-Whitney test was used. Correlation coefficients were calculated using Spearman’s rank method. Data are expressed as mean and dots represent individual patient values. * *P* < 0.05 ** *P* < 0.01.

**Additional file 6: Figure S6.** Autoreactive IgG and IgM does not correlate with disease progression. Fluorescence intensity for plasma autoreactive IgG (A) and IgM (B) does not correlate with decline in forced vital capacity (FVC) over 1-year period in IPF patients. Correlation coefficients were calculated using Spearman’s rank method.

**Additional file 7: Figure S7.** Gating strategy for B-cell subsets in mice. Representative gating strategy used for mice experiments for the identification of GC B-cells (CD19^+^CD95^+^IgD^low^), IgA GC B-cells (CD19^+^CD95^+^IgD^low^IgA^+^), plasma cells (CD19^low^CD138^+^) and IgA^+^ plasma cells (CD19^low^CD138^+^IgA^+^).

**Additional file 8: Figure S8.** Increased proportions of IgA^+^ GC B-cells and IgA^+^ plasma cells in lungs in mice exposed to saline and bleomycin. (A) Flow cytometric quantification of IgA^+^ GC B-cells (CD19^+^CD95^+^IgD^low^IgA^+^) as percentage of GC B-cells and (B) IgA^+^ plasma cells (CD19^low^CD138^+^IgA^+^) as percentage of plasma cells in lungs of mice exposed to saline or bleomycin. Data are expressed as mean and dots represent individual patient values. Nonparametric two-tailed Mann-Whitney test was used. * P < 0.05

**Additional file 9:.** Graphic summary of B-cell subset alterations and autoreactive IgA induction in IPF patients. Immature B cells leave the bone marrow as transitional B-cells for further differentiation. Decrease in circulating transitional B-cells together with their increase in IPF lungs suggest homing towards pulmonary tertiary lymphoid organs (TLO). Increased BTK levels in (immature) B-cells hinders adequate elimination of autoreactive B-cells to (pulmonary) self-antigens and might contribute to the development of autoreactive B-cells in pulmonary TLOs after epithelial injury. Furthermore, activated (PD-1 high) Tfh-cells are present in IPF lungs and they engage with B-cells in the GC of pulmonary TLOs, possibly further promoting autoreactive B-cell development. Cytokines produced by Tfh-cells and the local environment in IPF lungs induces predominantly IgA class-switch recombination. Upon activation, IgA memory B-cells and plasmablast leave pulmonary TLO leading to an increase of IgA memory B-cells, plasmablasts and free (autoreactive) IgA in blood of IPF patients.

**Additional file 10: Table S1.** Overview of antibodies used for experiments.

**Additional file 11: Methods S1.** Procedures of mouse experiments.

## Abbreviations

ANA: Antinuclear antibody
BAFF: B-cell activating factor
BALF: Broncho alveolar lavage fluid
BCR: B-cell receptor
CXCL: CXC chemokine ligand
DN: Double negative
EDTA: Ethylenediaminetetraacetic acid
FVC: Forced vital capacity
HC: Healthy control
HEp-2: Human epithelial cells
IPF: Idiopathic pulmonary fibrosis
LN: Lymph Node
MFI: Mean fluorescence intensity
PBS: Phosphate buffered saline
RPMI: Roswell Park Memorial Institute
Tfh: Follicular helper T-cells
TFS: Total fibrosis score
TLO: Tertiary lymphoid organ
