# Supplementary figures and images for: Enhanced Bruton’s tyrosine kinase in B-cells and autoreactive IgA in patients with idiopathic pulmonary fibrosis
Source: Respir Res. 2019 Oct 24;20:232. doi: 10.1186/s12931-019-1195-7 (PMC6814043; doi:10.1186/s12931-019-1195-7)

Blood

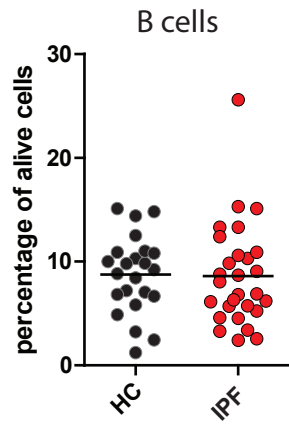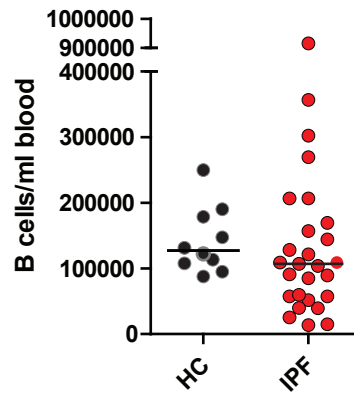

Lung

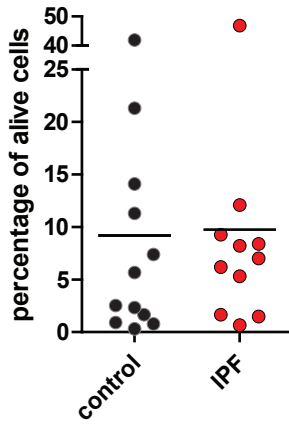

Lymph Node

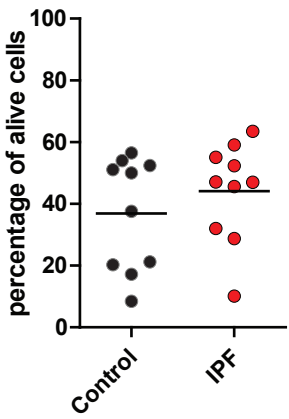

Supplement: Supplementary file 1 — Additional file 1: Figure S1. No changes of proportions of total B-cells between controls and IPF patients. Flow cytometric quantification of total B-cells (CD19+) in blood, lungs and lymph nodes (LN) as percentage of alive cells. For blood samples data also depicted as absolute number of B-cells per ml blood. Data are expressed as mean and dots represent individual values [file 12931_2019_1195_MOESM1_ESM.pdf]

Lymph  
Node

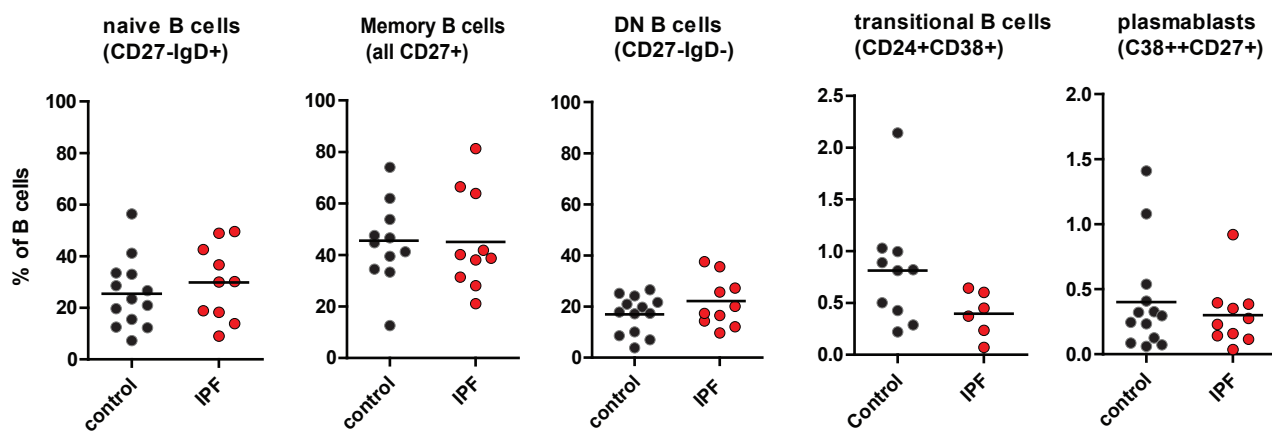

Supplement: Supplementary file 2 — Additional file 2: Figure S2. No alterations in B-cell subsets between control lymph nodes (LN) and IPF LN. Flow cytometric quantification of naïve B-cells (CD19+IgD+CD27-) IgD+ and IgD− memory CD27+ B-cells, double negative (DN) B-cells (CD19+IgD−CD27−), transitional B-cells (CD19+CD24+CD38+), and plasmablast (CD19+CD38+CD27+) as percentage of alive cells. Data are expressed as mean and dots represent individual values. [file 12931_2019_1195_MOESM2_ESM.pdf]

A

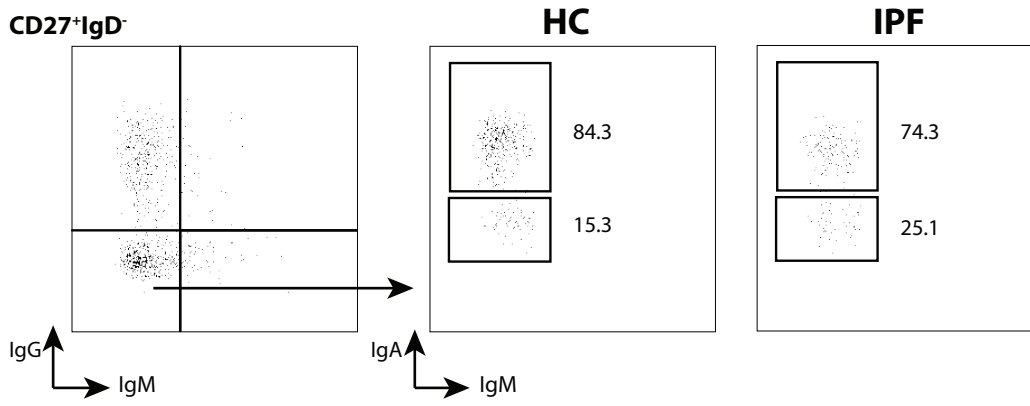

B

### CD27<sup>+</sup> Memory B cells

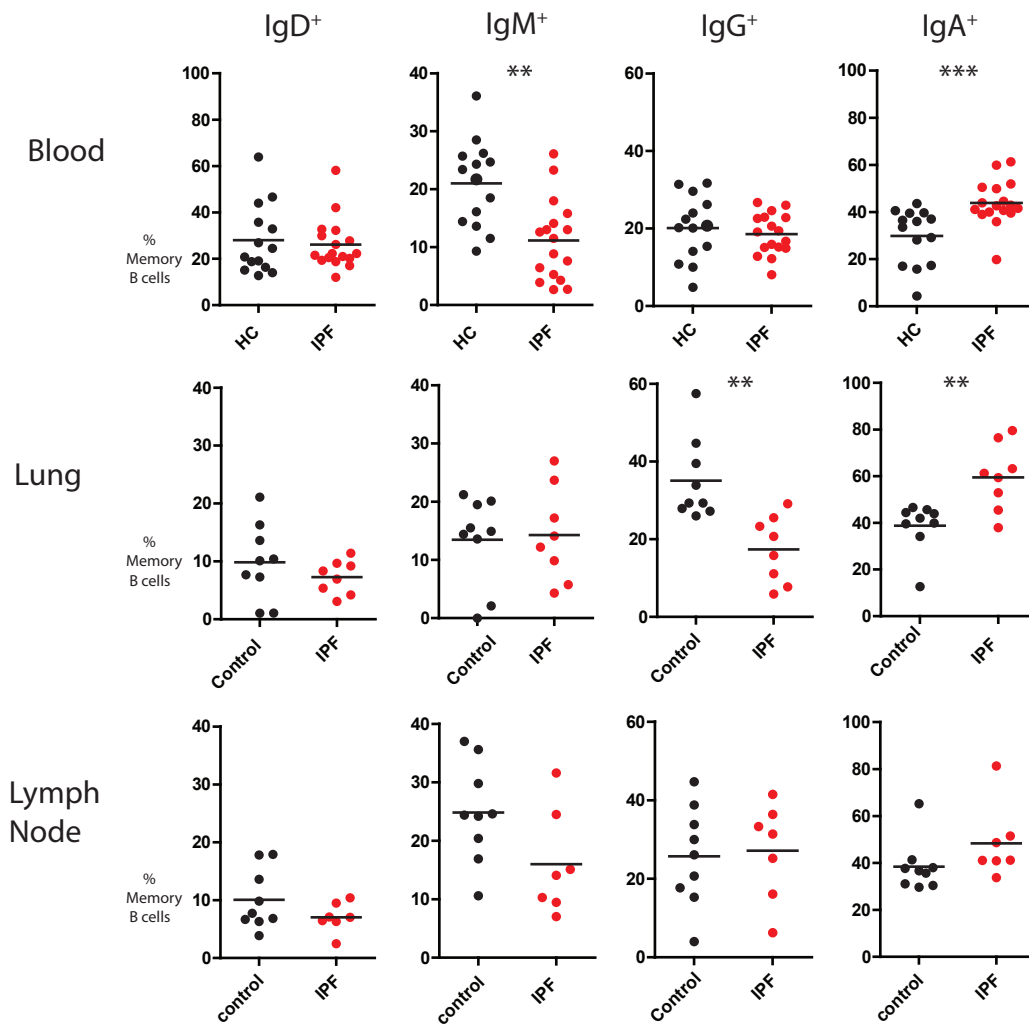

Supplement: Supplementary file 3 — Additional file 3: Figure S3. Augmented proportions of IgA-expressing CD27+ memory B-cell subsets. (A) Representative gating strategy for the identification of surface IgA expression on CD27+IgD− memory B-cells. The CD27+IgD−IgM−IgG− memory cells are enriched for IgA+ surface expression as depicted for a healthy control (HC) and IPF patient. (B) Flow cytometric analysis of the distribution of surface expression of IgD, IgM, IgG and IgA on CD27+ memory B-cells for blood, lungs and lymph nodes for controls and IPF patients. Non-parametric two-tailed Mann-Whitney test was used. ** P < 0.01 *** P < 0.001 [file 12931_2019_1195_MOESM3_ESM.pdf]

A

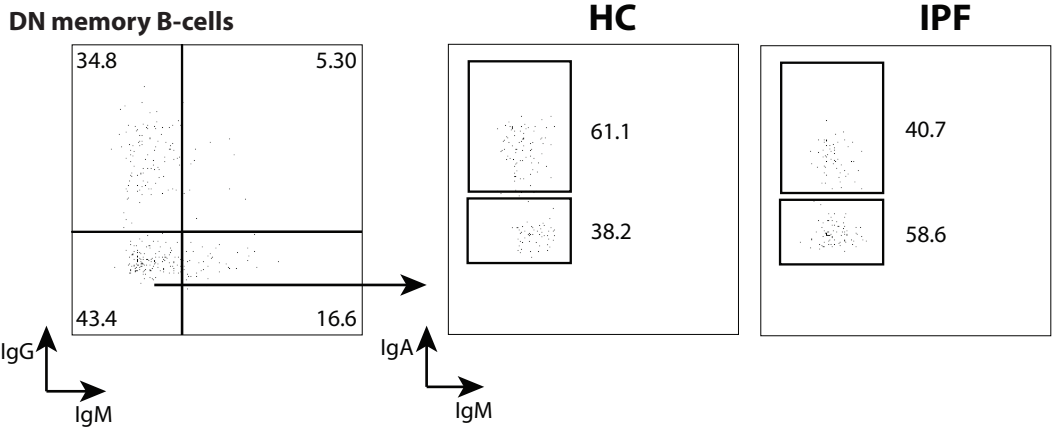

B

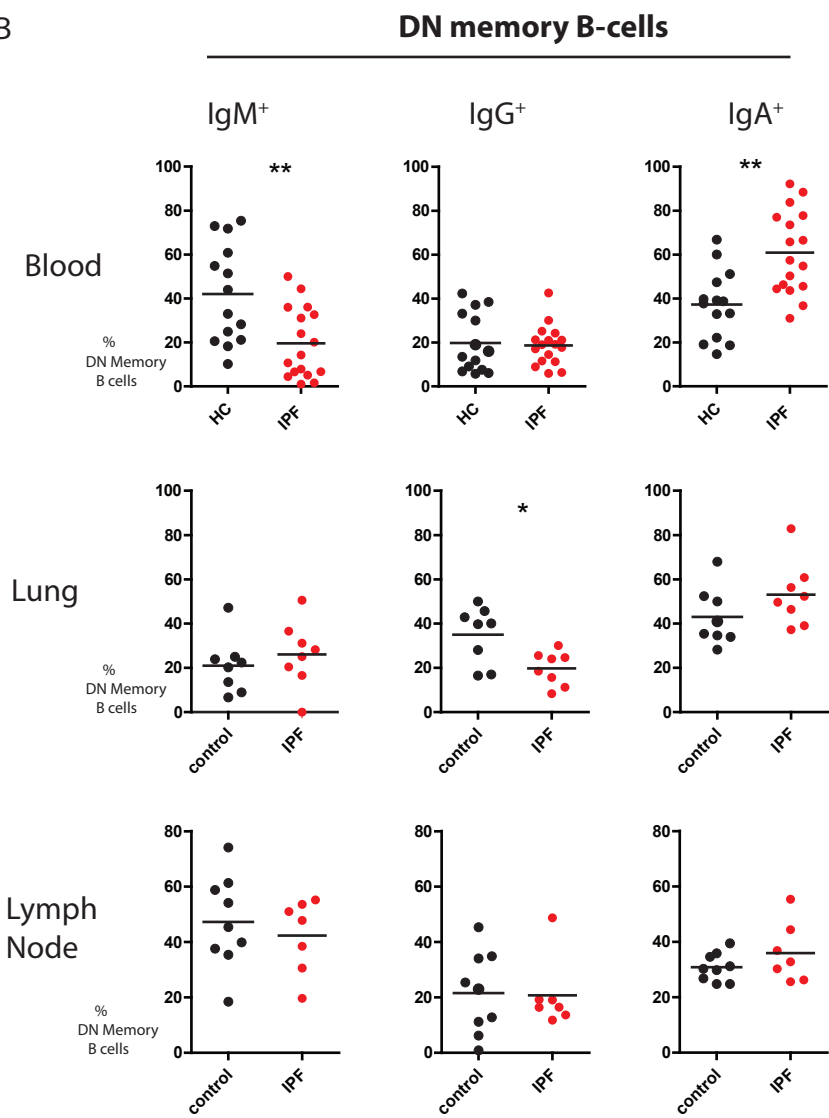

Supplement: Supplementary file 4 — Additional file 4: Figure S4. Augmented proportions of IgA-expressing DN memory B-cell subsets. (A) Representative gating strategy for the identification of surface IgA expression on double negative (DN) memory B-cells (CD19+CD27−IgD−). The IgM−IgG− DN memory cells are enriched for IgA+ surface expression as depicted for a healthy control (HC) and IPF patient. (B) Flow cytometric analysis of the distribution of surface expression of IgM, IgG and IgA on DN memory B-cells for blood, lungs and lymph nodes for controls and IPF patients. Non-parametric two-tailed Mann-Whitney test was used. ** P < 0.01 *** P < 0.001 [file 12931_2019_1195_MOESM4_ESM.pdf]

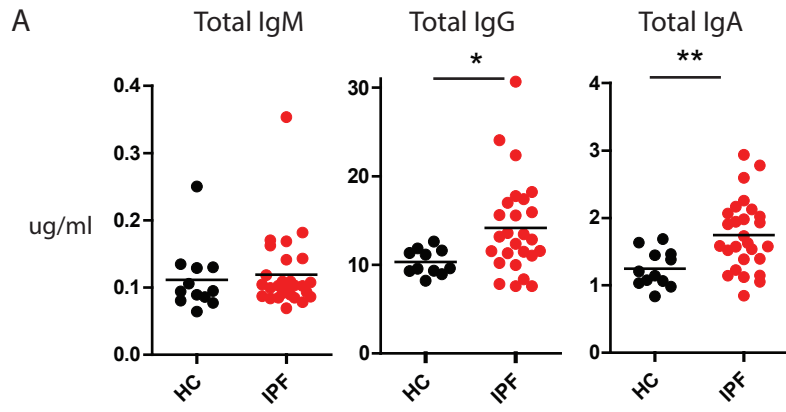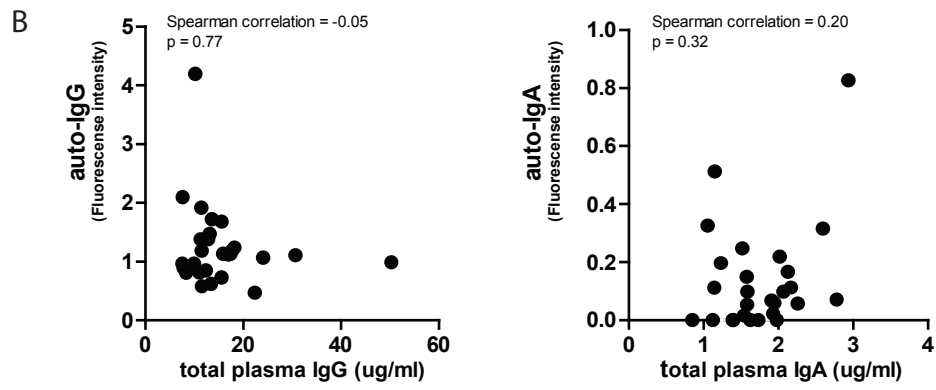

Supplement: Supplementary file 5 — Additional file 5: Figure S5. Increased total IgG and IgA in plasma of IPF patients. (A) total plasma IgM, IgG and IgA levels (μg/ml) for HC and IPF patients. (B) Correlation between total IgG or IgA (μg/ml) and autoreactive IgG or IgA. Indirect quantification of auto-reactive immunoglobulin levels depicted as fluorescence intensity measured on HEp-2 slides. Non-parametric two-tailed Mann-Whitney test was used. Correlation coefficients were calculated using Spearman’s rank method. Data are expressed as mean and dots represent individual patient values. * P < 0.05 ** P < 0.01. [file 12931_2019_1195_MOESM5_ESM.pdf]

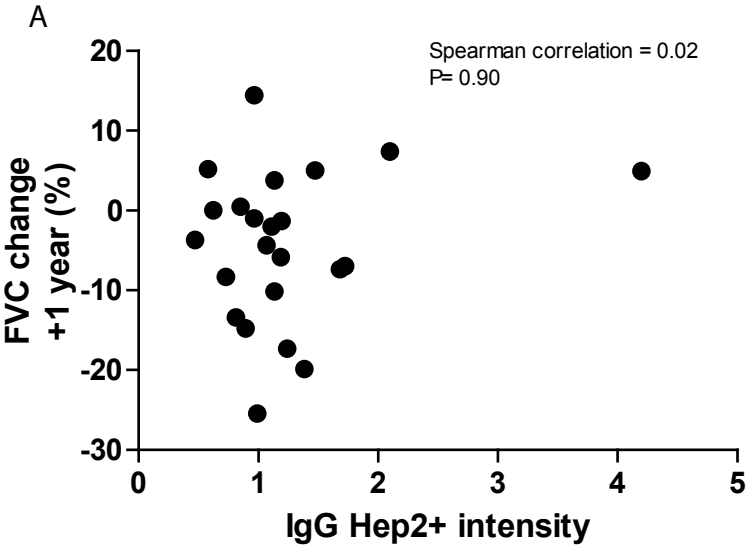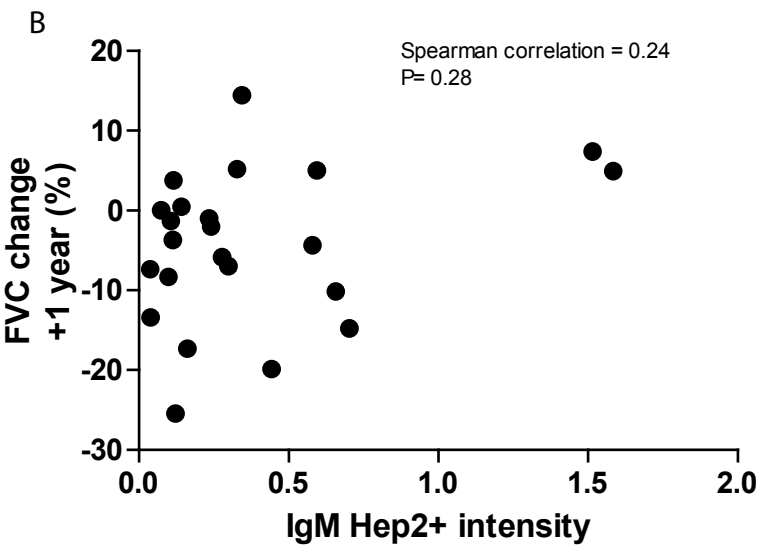

Supplement: Supplementary file 6 — Additional file 6: Figure S6. Autoreactive IgG and IgM does not correlate with disease progression. Fluorescence intensity for plasma autoreactive IgG (A) and IgM (B) does not correlate with decline in forced vital capacity (FVC) over 1-year period in IPF patients. Correlation coefficients were calculated using Spearman’s rank method. [file 12931_2019_1195_MOESM6_ESM.pdf]

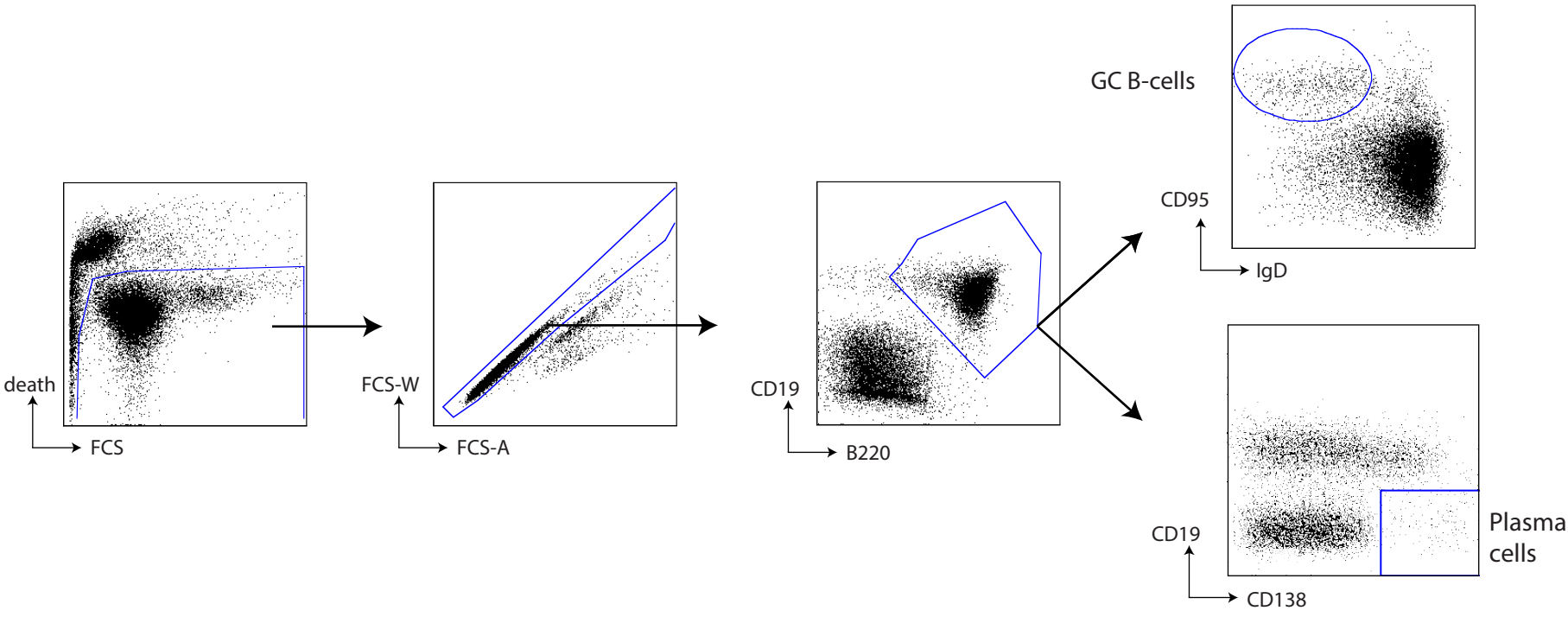

Lorem ipsum

Supplement: Supplementary file 7 — Additional file 7: Figure S7. Gating strategy for B-cell subsets in mice. Representative gating strategy used for mice experiments for the identification of GC B-cells (CD19+CD95+IgDlow), IgA GC B-cells (CD19+CD95+IgDlowIgA+), plasma cells (CD19lowCD138+) and IgA+ plasma cells (CD19lowCD138+IgA+). [file 12931_2019_1195_MOESM7_ESM.pdf]

A

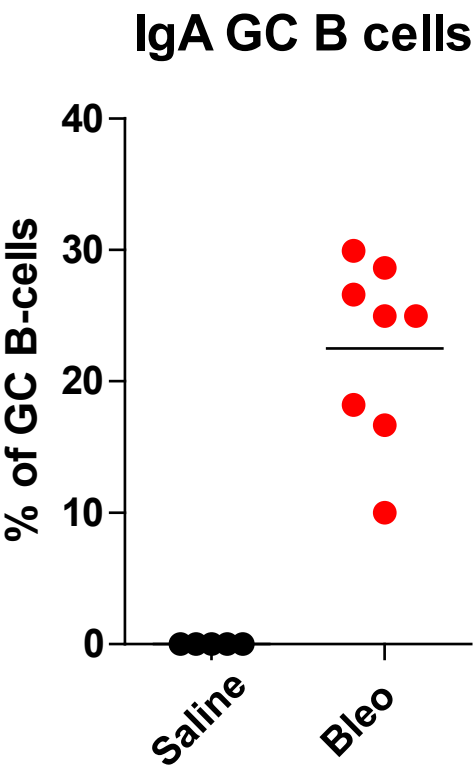

B

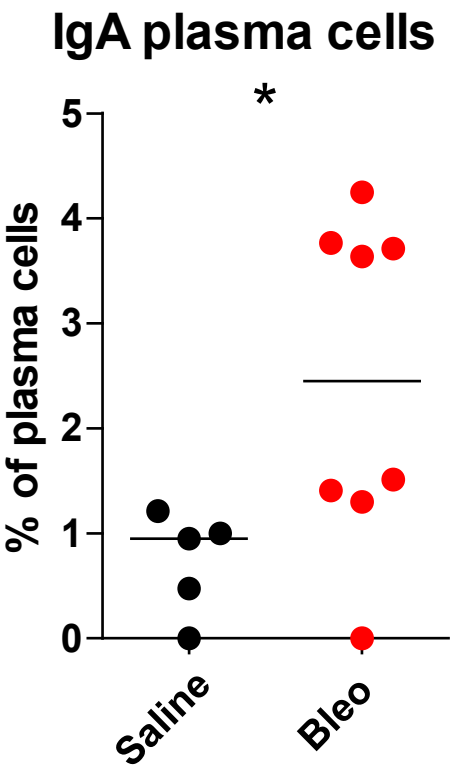

Supplement: Supplementary file 8 — Additional file 8: Figure S8. Increased proportions of IgA+ GC B-cells and IgA+ plasma cells in lungs in mice exposed to saline and bleomycin. (A) Flow cytometric quantification of IgA+ GC B-cells (CD19+CD95+IgDlowIgA+) as percentage of GC B-cells and (B) IgA+ plasma cells (CD19lowCD138+IgA+) as percentage of plasma cells in lungs of mice exposed to saline or bleomycin. Data are expressed as mean and dots represent individual patient values. Nonparametric two-tailed Mann-Whitney test was used. * P < 0.05 [file 12931_2019_1195_MOESM8_ESM.pdf]

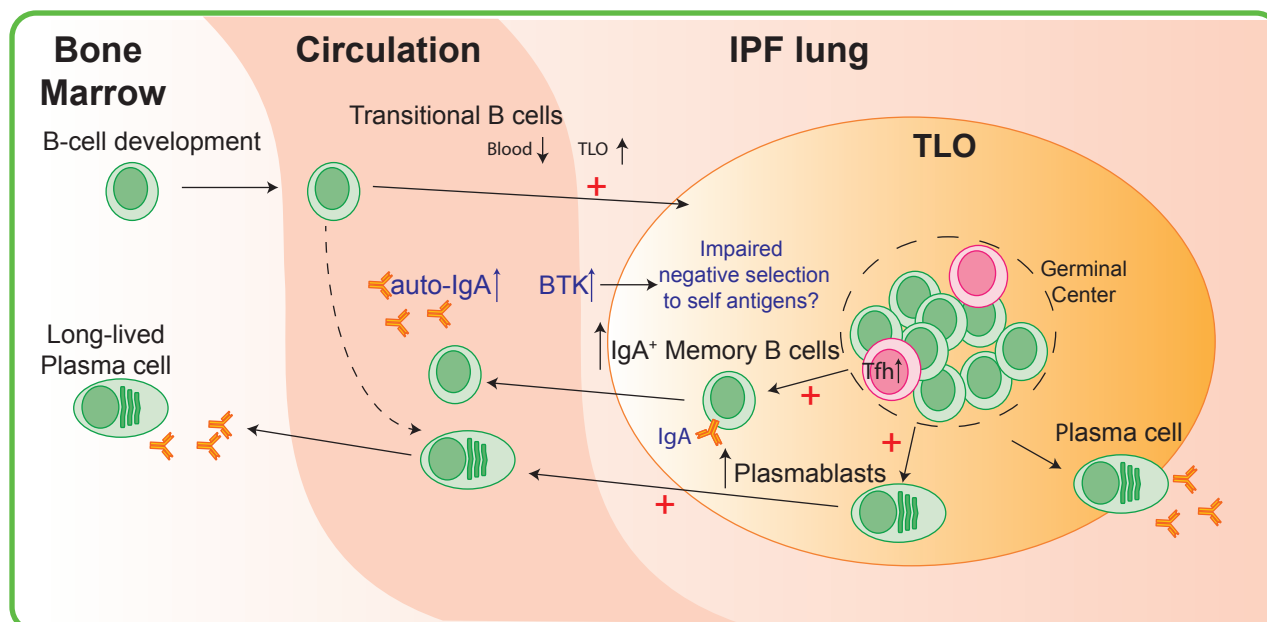

Supplement: Supplementary file 9 — Additional file 9:. Graphic summary of B-cell subset alterations and autoreactive IgA induction in IPF patients. Immature B cells leave the bone marrow as transitional B-cells for further differentiation. Decrease in circulating transitional B-cells together with their increase in IPF lungs suggest homing towards pulmonary tertiary lymphoid organs (TLO). Increased BTK levels in (immature) B-cells hinders adequate elimination of autoreactive B-cells to (pulmonary) self-antigens and might contribute to the development of autoreactive B-cells in pulmonary TLOs after epithelial injury. Furthermore, activated (PD-1 high) Tfh-cells are present in IPF lungs and they engage with B-cells in the GC of pulmonary TLOs, possibly further promoting autoreactive B-cell development. Cytokines produced by Tfh-cells and the local environment in IPF lungs induces predominantly IgA class-switch recombination. Upon activation, IgA memory B-cells and plasmablast leave pulmonary TLO leading to an increase of IgA memory B-cells, plasmablasts and free (autoreactive) IgA in blood of IPF patients. [file 12931_2019_1195_MOESM9_ESM.pdf]
