# Supplementary material for: Enhanced Bruton’s tyrosine kinase in B-cells and autoreactive IgA in patients with idiopathic pulmonary fibrosis
Source: Respir Res. 2019 Oct 24;20:232. doi: 10.1186/s12931-019-1195-7 (PMC6814043; doi:10.1186/s12931-019-1195-7)
Supplement: Supplementary file 10 — Additional file 10: Table S1. Overview of antibodies used for experiments. [file 12931_2019_1195_MOESM10_ESM.docx]

**Additional tabel S1**

| marker | **conjugate** | **company** | **Cat#** | **intra/extracellular** | **dilution** |
| --- | --- | --- | --- | --- | --- |
| HUMAN |  |  |  |  |  |
|  |  |  |  |  |  |
| antibodies used for flow cytometry |  |  |  |  |  |
| IgG | FITC | BD | 555786 | extracellular | 1:20 |
| Btk | PE | BD | 611117 | intracellular | 1:5 |
| IgM | Bio | BD | 555781 | extracellular | 1:20 |
| CD19 | PerCP-Cy5.5 | BD | 332780 | extracellular | 1:400 |
| CD38 | APC | BD | 560980 | extracellular | 1:10 |
| IgD | APC-H7 | BD | 561305 | extracellular | 1:10 |
| CD27 | BV421 | BD | 562513 | extracellular | 1:80 |
| CD24 | BV711 | BD | 563401 | extracellular | 1:40 |
| CD3 | AF700 | eBioscience | 56-0038-42 | extracellular | 1:40 |
| CD33 | PeCy7 | eBioscience | E10580-351 | extracellular | 1:30 |
| IgA | PE | BD | 555935 | extracellular | 1:10 |
| CXCR5 | PerCP5.5 | BD | 562781 | extracellular | 1:20 |
| CD3 | APC ef780 | eBioscience | 47-0038-42 | extracellular | 1:100 |
| CD4 | AF700 | eBioscience | E08948-1631 | extracellular | 1:100 |
| CD45RA | BV650 | BD | 563963 | extracellular | 1:40 |
| PD1 | BV786 | BD | 563789 | extracellular | 1:20 |
| FoxP3 | PE | eBioscience | 12-4777-42 | intracellular | 1:20 |
|  |  |  |  |  |  |
| Immunohisto- chemistry Lung |  |  |  |  |  |
| CD3 |  | Ventana | Clone: 2GV6 |  | ready to use |
| CD20 |  | Ventana | Clone: L26 |  | ready to use |
| CD79 |  | Ventana | Clone: SP18 |  | ready to use |
| IgA |  | Cell Marque | Rabbit Polyclonal |  | ready to use |
| IgG |  | DAKO | Rabbit Polyclonal |  | 1:24000 |
|  |  |  |  |  |  |
| HEp-2 antibodies |  |  |  |  |  |
| IgG | Cy3 | Jackson IR | 109-166-003 |  |  |
| IgM | af488 | Jackson IR | 109-546-129 |  |  |
| IgA | af647 | Jackson IR | 109-606-011 |  |  |
|  |  |  |  |  |  |
|  |  |  |  |  |  |
|  |  |  |  |  |  |
| MOUSE |  |  |  |  |  |
| antibodies used for flow cytometry |  |  |  |  |  |
| GL7 | FITC | BD | 553666 | extracellular | 1:2000 |
| CD95 | PE-TxR | BD | 562499 | extracellular | 1:400 |
| IgM | Pe-Cy7 | eBioscience | 25-5790-82 | extracellular/  intracellular | 1:500 |
| IgD | APC | eBioscience | 17-5993-82 | extracellular/  intracellular | 1:1280 |
| CD19 | Af700 | eBioscience | 56-0193-82 | extracellular | 1:50 |
| CD138 | BV605 | BD | 563147 | extracellular | 1:400 |
| CD3 | PE-CF594 | BD | 562286 | extracellular | 1:100 |
| CD4 | Af700 | eBioscience | 56-0041-82 | extracellular | 1:200 |
| CD4 | Af700 | eBioscience | 56-0041-82 | extracellular | 1:400 |
| IgA | FITC | BD | 559354 | intracellular | 1:200 |
| Immunohisto- chemistry Lung |  |  |  |  |  |
| GL-7 | FITS | BD | 3036700 |  | 1:50 |
| IgD | PE | eBioscience | E02008-1634 |  | 1:50 |
|  |  |  |  |  |  |
|  |  |  |  |  |  |
