# Supplementary material for: Enhanced Bruton’s tyrosine kinase in B-cells and autoreactive IgA in patients with idiopathic pulmonary fibrosis
Source: Respir Res. 2019 Oct 24;20:232. doi: 10.1186/s12931-019-1195-7 (PMC6814043; doi:10.1186/s12931-019-1195-7)
Supplement: Supplementary file 11 — Additional file 11: Methods S1. Procedures of mouse experiments. [file 12931_2019_1195_MOESM11_ESM.docx]

**additional methods 1**

**Inducing pulmonary fibrosis (mice)**

To induce pulmonary fibrosis, bleomycin-hydrochloride was administered intratracheally in 8-10 week old mice (0,04U/80 μl saline) or saline as a control.^1^ Mice were sacrificed 21 days after bleomycin exposure.

**Flow cytometic procedures (mice)**

Preparations of single-cell suspensions using standard procedures. Monoclonal antibodies are listed in additional table 1. For intracellular staining, cells were fixed in Cytofix/Cytoperm and permeabilized, and then stained in Perm/Wash buffer (BD Bioscience). All measurements were performed on a LSRII flow cytometer (BD Bioscience), and results were analyzed using FlowJo software.

**Immunohistochemistry (mice)**

Immunohistochemical analyses and staining were performed according to standard procedures.^2^ Used antibodies are listed in additional table 1. After staining, tissue sections were embedded in Kaiser glycerol gelatin (Merck). Micrographs were made using a DM LB light microscope (Leica), a DFC500 camera (Leica), and Imaging for Windows Version 1.0 software (Kodak). A pathologist (blinded for treatment) scored the Ashcroft scale (grade 1-8)^3^ and the percentage of lung involvement (grade 1-5; 1 =0-10% to 6 = 75-100% of total lung involvement). The Total Fibrosis score (TFS) is the product of Ashcroft scale and lung involvement and was previously described.^4^

**References**

1. Kim SN, Lee J, Yang HS, Cho JW, Kwon S, Kim YB*, et al.* Dose-response Effects of Bleomycin on Inflammation and Pulmonary Fibrosis in Mice. *Toxicol Res* 2010, **26**(3)**:** 217-222.

2. GeurtsvanKessel CH, Willart MA, Bergen IM, van Rijt LS, Muskens F, Elewaut D*, et al.* Dendritic cells are crucial for maintenance of tertiary lymphoid structures in the lung of influenza virus-infected mice. *J Exp Med* 2009, **206**(11)**:** 2339-2349.

3. Ashcroft T, Simpson JM, Timbrell V. Simple method of estimating severity of pulmonary fibrosis on a numerical scale. *J Clin Pathol* 1988, **41**(4)**:** 467-470.

4. Borensztajn K, Bresser P, van der Loos C, Bot I, van den Blink B, den Bakker MA*, et al.* Protease-activated receptor-2 induces myofibroblast differentiation and tissue factor up-regulation during bleomycin-induced lung injury: potential role in pulmonary fibrosis. *Am J Pathol* 2010, **177**(6)**:** 2753-2764.
